# The cardioprotective and antiarrhythmic effects of *Nardostachys chinensis* in animal and cell experiments

**DOI:** 10.1186/s12906-017-1910-1

**Published:** 2017-08-10

**Authors:** Min Li, Xue Xu, Xinyu Yang, Joey S. W. Kwong, Hongcai Shang

**Affiliations:** 1 Key laboratory of Chinese Internal Medicine of Ministry of Education and Beijing, Dongzhimen Hospital, Beijing University of Chinese Medicine, Beijing, China; 2 Beijing University of Chinese Medicine, Beijing, China; 3 Department of Clinical Epidemiology and Department of Health Policy, National Centre for Child Health and Development, Tokyo, Japan; 4 Institute of Integration of Traditional Chinese and Western Medicine, Guangzhou Medical University, Guangzhou, China

**Keywords:** *Nardostachys chinensis*, NC, Cardioprotective effects, Antiarrhythmic effects, Animal experiments, Cell experiments

## Abstract

**Background:**

Cardiovascular disease (CVD) is the leading cause of premature death throughout the world. An estimated 17.5 million people died from CVD in 2012, representing 31% of all global deaths. *Nardostachys chinensis* (NC), a typical traditional Chinese medicine (TCM), plays a crucial role in the management of patients with CVD, especially for those with cardiac arrhythmia. The purpose of this study was to evaluate the cardioprotective and antiarrhythmic effects of NC in animal and cell experiments.

**Methods:**

To review the cardioprotective and antiarrhythmic effects of NC, studies of NC on cardiovascular diseases in animal and cell experiments were identified from five databases through April 2016. Two investigators independently conducted the literature search, study selection, and data extraction.

**Results:**

A total of 16 studies were identified, including five animal experiments and eleven cell experiments. Four studies showed significant effects of NC on myocardial protection by inhibiting myocardial apoptosis, inflammation and oxidative stress. Twelve studies indicated significant beneficial effects of NC in cardiac arrhythmia primarily through the modulation of ion channels (I_k_, I_k1_, I_Na_, I_Ca-L_, I_to_).

**Conclusion:**

The above findings showed the possible efficacy of NC via its cardioprotective and antiarrhythmic effects, but the results should be interpreted with caution due to the limitations and the deficiencies in the studies.

## Background

Cardiovascular disease (CVD) is the leading cause of premature death throughout the world. An estimated 17.5 million people died from CVD in 2012, representing 31% of all global deaths [1]. Cardiac arrhythmias, abnormalities in the heart rate or rhythm, are a form of CVD that affect the pumping function of the heart [2, 3]. The electrophysiological mechanism of cardiac arrhythmia mainly involves two aspects, disorders of impulse conduction (triggered activity, re-entry) and anomalies of electric impulse formation (mostly enhanced automaticity) [4]. Based on these mechanisms, multiple therapeutic measures, such as pharmacological treatment, electric defibrillation, radiofrequency catheter ablation, and artificial cardiac pacing, are extensively developed and employed in the treatment of cardiac arrhythmias [5, 6]. In most cases, pharmacological interventions are the preferred therapeutic method. Unfortunately, almost all western antiarrhythmic drugs are associated with notable adverse effects, and certain agents may even be proarrhythmic [7] as confirmed by the Cardiac Arrhythmia Suppression Trial (CAST) [8]. Due to the limitations of the currently available treatments, complementary and alternative medicine (CAM) is increasingly sought to treat cardiac arrhythmia. Traditional Chinese medicine (TCM), a major type of CAM, has been used in patients with cardiac arrhythmia for thousands of years and remains a popular treatment option in China and worldwide [9]. However, studies on the mechanisms of TCM in the treatment of cardiac arrhythmia are lacking, and further exploration is greatly needed.

*Nardostachys chinensis* (NC), the rhizomes and roots of *Nardostachys jatamansi DC*, can rectify qi, relieve pain, resolve constraint and fortify the spleen according to TCM theory [10]. NC has a long history of usage as an ethnomedicine and has been used to treat various diseases of different systems, such as indigestion, vomiting, hyperglycaemia, epilepsy, hysteria, and dyslipidaemia [11, 12]. The currently available data suggest the application of NC in CVD, especially in cardiac arrhythmia [13, 14]. Meanwhile, the Chinese patent medicines Wenxin Keli and Shensong Yangxin capsule play crucial roles in the treatment of all types of cardiac arrhythmias, e.g., ventricular premature beat (VPB), atrial fibrillation, and supraventricular arrhythmia [15–17]. However, little is known about the mechanism of NC in CVD from animal and cell experiments. Consequently, we conducted this systematic review to evaluate the cardioprotective and antiarrhythmic effects of NC in animal and cell experiments and provided some clues for researchers to develop new antiarrhythmic drugs based on NC.

## Methods

### The goal

The purpose of this study is to evaluate the cardioprotective and antiarrhythmic effects of NC in animal and cell experiments.

### Search methods for the identification of studies

PubMed, The Cochrane Library, China National Knowledge Infrastructure (CNKI), the Wanfang Database, and the Chinese Scientific Journal Database (VIP) were searched for eligible studies from inception to April 2016. The search strategy used the following general terms: “*Nardostachys chinensis*”, “*Nardostachys chinensis Batal*”, “*Nardostachys jatamansi DC*”, “*Nardosinone*”, “*Nardostahyos Radix et Rhizoma*”, “Animal”, “Mouse”, “Mice”, “Rat”, “Rabbit”, “Pig”, “Swine”, “Hog”, “Sheep”, “Dog”, “Monkey”, “Cat”, “Ape”, “Hominoidea”, and “cell”.

### Criteria for the consideration of studies for this review

Studies on the cardiovascular system in animal and cell experiments were considered for inclusion in this review. The intervention was a single medicine, *Nardostachys chinensis*, or its active compounds. Studies on proprietary Chinese medicines and traditional Chinese medicine decoctions were excluded, though NC was one of their main ingredients. We included only journal articles and academic dissertations.

### Data extraction and management

Two investigators independently conducted the literature search, study selection, and data extraction. The data from the included studies were managed using a pre-standardized extraction form. The extracted data include the study name, the type of experiment, the intervention, the dose, groups, randomness, outcomes and so on. Disagreements were discussed and resolved in a consensus meeting with the corresponding author.

### Assessment of the risk of bias for animal studies

According to SYRCLE’s risk of bias tool, we assessed the animal studies [18].

## Results

### Search process

The initial search using the electronic search strategies yielded 169 studies. After removing 47 duplicates from different databases, we evaluated 122 potentially relevant articles for eligibility. After screening the titles and abstracts, we excluded 101 studies. Of the 21 remaining studies, we further excluded 5 studies after screening the full-text articles. Eventually, we included 16 studies. A flow chart (Fig. 1) illustrates our search process and study selection.

### Included studies

We included 16 studies in this review [19–34]. Among them, 12 studies were published in Chinese journals, [19, 23, 24, 26–34] and 4 studies were published in English-language journals [20–22, 25]. Five studies were associated with animal experiments [19, 20, 23–25], and 11 studies were related to cell experiments [21, 22, 26–34]. 4 studies reflected the reported cardioprotective effects of NC [19–22], including 2 studies based on cell experiments [19, 20] and 2 studies based on animal experiments [21, 22]. 12 studies showed the antiarrhythmic effects of NC [23–34], including 9 studies based on cell experiments [26–34] and 3 studies based on animal experiments [23–25].

### The cardioprotective effects of NC

Two cell experiments showed that the active compounds of NC had cardioprotective effects [21, 22]. One intervention used the volatile oil of NC [21], and the other used Nardosinone [22]. They both verified the myocardial protective effect of NC in cultured H9c2 cardiomyocytes. Oxidative stress was a basic cause of cardiovascular diseases [35]. Reactive oxygen species (ROS), the major product of oxidative stress, can lead to cardiomyocyte dysfunction, I/R injury, and heart failure [36]. Tert-butyl hydroperoxide (tBHP) can cause an accumulation of excess intracellular ROS and induce H9c2 cardiomyocyte injury and even death. When H9c2 cells were pre-treated with the volatile oil of NC before the addition of tBHP, cell survival increased in a dose-dependent manner [21]. A high dose of volatile oil almost fully protected the cells from injury. Under a light microscope, the volatile oil-treated H9c2 cardiomyocytes remained healthy even after a tBHP challenge, and the control cells shrank. In addition, the volatile oil of NC reduced the ROS level and induced antioxidant response element (ARE) transcriptional activity. The activation of the Akt signalling has been shown to exhibit a cardioprotective effect. The volatile oil of NC can activate the phosphorylation of Akt in H9c2 cardiomyocytes. Simultaneously, the cell protective effects of the volatile oil would weaken and even disappear when an Akt inhibitor was added. Cardiac hypertrophy was an effective and adaptive compensatory response under various stimuli or pressure loads. However, this kind of compensatory response may result in heart failure, cardiac arrhythmia or even sudden death with continuous development [37]. It has been shown that Angiotensin II (Ang II), as a vital mediator, could cause cardiac hypertrophy [38]. Nardosinone, one of main extracts of NC, could significantly inhibit the enlargement of the cell surface area induced by Ang II and the mRNA expressions of atrial natriuretic peptide (ANP), B-type natriuretic peptide (BNP) and β-myosin heavy chain (β-MHC) in a concentration- and time-dependent manner [22]. In addition, the protective effects of nardosinone may be mediated by repressing the phosphorylation of related proteins in the phosphatidylinositol 3-kinase (PI3K)/Serine-threonine kinase Akt pathway and the mitogen-activated protein kinase (MEK) /extracellular signal-regulated kinase (ERK) pathway.

Two animal experiments reported the cardioprotective effects of NC using the volatile oil and the extract of NC as interventions [19, 20] (Table 1). Acute myocardial infarction (AMI) is a common cardiovascular disease that causes serious damage to human health. The morbidity of AMI increased year by year, becoming a vital public health problem [39, 40]. Currently, one of the most effective therapeutic measures for patients with AMI has been the restoration of the blood supply [41]. However, sudden reperfusion after a relatively long period of ischaemia could result in Ischaemia- Reperfusion (I/R) injury [42]. To moderate this injury, many studies have concentrated on interventions that can protect the heart [43, 44]. The goal of the first animal experiment was to observe the effects of the volatile oil of NC at different time points on myocardial I/R injury in rats [19]. The sham group received ligature but not ligation on the anterior descending coronary artery. The I/R model group underwent ligation and ligature, which was released after 20 min followed by 60 min of reperfusion. The volatile oil of NC was given to the NC group-1 rats 15 min before ligation and to the NC group-2 rats 15 min before reperfusion. Compared with the I/R model group, NC group-1 and NC group-2 showed significantly reduced serum levels of Interleukin-6 (IL-6), Tumour Necrosis Factor (TNF-α), Creatine kinase-MB (CK-MB), cardiac troponin T (cTnT), left ventricular infarction size, and frequency and duration of ventricular arrhythmia. In view of the above outcomes, the results of the volatile oil of NC group-1 were more significant than those for group-2. The use of doxorubicin has been shown to cause oxidative damage, cardiomyopathy, and even congestive heart failure [45, 46]. In the second animal experiment, doxorubicin decreased the antioxidant enzymes superoxide dismutase [SOD], catalase [CAT], glutathione peroxidase [GPx], and glutathione-S- transferase [GST]) and increased the cardiac markers lactate dehydrogenase [LDH], creatine phosphokinase [CPK], aspartate aminotransaminase [AST], and alanine aminotransaminase [ALT]) compared with the controls [20]. In addition, lipid metabolism was also abnormal. NC has many active constituents such as sesquiterpene, lignan, neolignan, terpenoid, etc. Studies have indicated that these compounds in various plants possess antioxidant activity. Pretreatment with the extract of NC significantly restored enzyme activity, cardiac markers and lipid peroxides. These results demonstrated that NC has cardioprotective effects via the inhibition of the inflammatory reaction and myocardial injury.

**Table 1:** The detailed information of the animal experiments

Study	Species	Weight(g)	Random	Groups	Outcome measure
Tao Yang et al. 2012	SD rats	200 ± 20	Not mentioned	The sham group (*n* = 10) The I/R model group (*n* = 10) The volatile oil of NC group-1 (*n* = 10) The volatile oil of NC group-2 (*n* = 10)	IL-6, TNF-ɑ, CK-MB, cTNT, Infarct size, The frequency and time of cardiac arrhythmia
Rajakannu Subashini et al. 2006	Wistar rats	120–130	Not mentioned	The control group (*n* = 6) The doxorubicin group (*n* = 6) The extract of NC group (*n* = 6) The doxorubicin + extract of NC group (*n* = 6)	Body weight; Heart weight; LDH; CPK; AST; ALT; SOD; CAT; GPx; GST; LPO

*I/R* Ischaemia-Reperfusion, *IL-6* Interleukin-6, *TNF-ɑ* Tumour Necrosis Factor, *CK-MB* Creatine kinase-MB, *LDH* Lactate dehydrogenase, *CPK* Creatine phosphokinase, *AST* Aspartate aminotransaminase, *ALT* Alanine aminotransaminase, *SOD* Superoxide dismutase, *CAT* Catalase, *GPx* Glutathione peroxidase, *GST* Glutathione-S-transferase, *LPO* Lipid peroxide

### The antiarrhythmic effect of NC

Nine studies reported the antiarrhythmic effects of the volatile oil and the extract of NC in cell experiments with the whole-cell patch-clamp technique [26–34]. The patch-clamp technique is an electrophysiological technique for studying cells, cell membranes, and isolated organelles. All patch-clamp methods depend on a very high-resistance seal between a membrane and a micropipette to control the voltage and monitor the currents of ion channels [47]. One study reported a delayed rectifier potassium current (I_k_) and an inward rectifier potassium current (I_k1_) [26]. Two studies reported an L-type calcium current (I_Ca-L_) [27, 28] and a transient outward potassium current (I_to_) [29, 31]. The other studies reported on sodium currents (I_Na_) [27, 29, 30, 32–34]. The detailed information is given in Table 2.

**Table 2:** The detailed information of the cell experiments

Study	Cell	Intervention	Groups	Target	Outcome measure
Xiangyu Li et al. 2013	The normal isolated ventricular myocytes of rats	The volatile oil of NC	The volatile oil of NC group-1 (1 μg/g) The volatile oil of NC group-2 (3 μg/g) The volatile oil of NC group-3 (5 μg/g) The volatile oil of NC group-4 (10 μg/g) The volatile oil of NC group-5 (20 μg/g) The volatile oil of NC group-6 (50 μg/g)	I_k_; I_k1_	Dose-response; I-V curve; Activation curve; Inactivation curve
Qizhu Tang et al. 2004	The normal isolated ventricular myocytes of rabbits	The extract of NC	The control group The extract of NC group-1 (5 g/L) The extract of NC group-2 (10 g/L)	I_Na_; I_Ca-L_	Dose-response; I-V curves
Ming Cao et al. 2010	The normal isolated ventricular myocytes of rats	The volatile oil of NC	The volatile oil of NC group-1 (3 μg/g) The volatile oil of NC group-2 (5 μg/g) The volatile oil of NC group-3 (10 μg/g) The volatile oil of NC group-4 (20 μg/g) The volatile oil of NC group-5 (50 μg/g)	I_Ca-L_	Dose-response; I-V curve; Activation curve; Inactivation curve
Yuanwei Liu et al. 2009	The normal isolated ventricular myocytes of rats	The extract of NC	The control group The extract of NC group-2 (10 g/L)	I_Na_; I_to_	I-V curve
Tao Yang et al. 2009	The normal isolated ventricular myocytes of rats	The volatile oil of NC	The volatile oil of NC group-1 (1 μg/g) The volatile oil of NC group-2 (3 μg/g) The volatile oil of NC group-3 (5 μg/g) The volatile oil of NC group-4 (10 μg/g) The volatile oil of NC group-5 (100 μg/g)	I_Na_	Dose-response; I-V curve; Activation curve; Inactivation curve
Langjie Hu et al. 2009	The normal isolated ventricular myocytes of rats	The volatile oil of NC	The volatile oil of NC group-1 (3 μg/g) The volatile oil of NC group-2 (6 μg/g) The volatile oil of NC group-3 (10 μg/g) The volatile oil of NC group-4 (20 μg/g)	I_to_	Dose-response; I-V curve; Activation curve; Inactivation curve
Yuzhi Ge et al. 2009	Na_v_ 1.5-HEK cell	The volatile oil of NC	The volatile oil of NC group-1 (3 ppm) The volatile oil of NC group-2 (10 ppm)	I_Na_	Dose-response; Voltage-dependent curve
Zhiting Wu et al. 2009	Na_v_ 1.5-HEK cell	The volatile oil of NC	The volatile oil of NC group (10 ppm)	I_Na_	I-V curve; G-V curve
Yanyang Liu et al. 2013	Na_v_ 1.5-HEK cell	The volatile oil of NC	The volatile oil of NC group (5 ppm)	I_Na_	Frequency-dependent curve

*I-V* Current-voltage relationship, *G-V* Conductance-voltage relationship

Six studies with eight groups showed the dose-responses between the active compounds of NC and the inhibition ratio of a current at a fixed voltage [26–31]. Two studies described dose-responses, but no detailed inhibition ratio information was provided [27, 29]. The other four studies with five groups gave detailed descriptions and used the volatile oil of NC as the intervention [26, 28, 30, 31]. The inhibition ratio of the current was closely related to the dose of the volatile oil. Generally, the current inhibition ratio would increase gradually as the dose of the volatile oil increased. Further details are given in Table 3.

**Table 3:** The information of dose-response

Study	Xiangyu Li et al. 2013	Xiangyu Li et al. 2013	Ming Cao et al. 2010	Tao Yang et al. 2009	Langjie Hu et al. 2009
Type	I_k_	I_k1_	I_Ca-L_	I_Na_	I_to_
Inhib-ition Ratio	1 μg/g (10.45 ± 1.14)% 3 μg/g (20.18 ± 2.54)% 5 μg/g (46.88 ± 5.30)% 10 μg/g (78.00 ± 9.20)% 20 μg/g (96.00 ± 6.40)% 50 μg/g (98.00 ± 5.60)%	1 μg/g (5.78 ± 1.32)% 3 μg/g (12.45 ± 1.14)% 5 μg/g (29.18 ± 2.44)% 10 μg/g (52.43 ± 6.3)% 20 μg/g(89.74 ± 11.3)% 50 μg/g (98.96 ± 4.6)%	3 μg/g (11.18 ± 1.48)% 5 μg/g (24.83 ± 4.82)% 10 μg/g (45.72 ± 3.54)% 20 μg/g (76.87 ± 7.03)% 50 μg/g (100 ± 0)%	1 μg/g (7.2 ± 4)% 3 μg/g (20 ± 3.7)% 5 μg/g (55 ± 5)% 10 μg/g (76 ± 3.5)% 100 μg/g (89 ± 3)%	3 μg/g (27.01 ± 6.93)% 6 μg/g (51.13 ± 9.82)% 10 μg/g (80.86 ± 4.63)% 20 μg/g (94.81 ± 4.30)%

Seven studies described the current density and voltage (I-V) curve [26–32]. I-V represented interactions in ion channels and an I-V curve was usually drawn when voltage and current density were taken as the abscissa and the ordinate. Thus, if voltage changed, the current density would also change without any interventions. Compared with a control group, the volatile oil or the extract of NC could decrease current density under the corresponding voltage. However, this decrease in current density was reversible. Generally, the current density of isolated cells would rise and even completely recover when these cells were washed with extracellular fluid.

The activation or inactivation of some ion channels depends on membrane voltage. Four studies reported the activation and inactivation of ion channels through half of the activating voltage (V_1/2_) and the slope factor according to the Boltzmann Equation [26, 28, 30, 31]. The volatile oil of NC had a negative effect on the activation of ion channels by acting on membrane voltage and exhibited a positive effect on inactivation. The details are given in Tables 4 and 5.

**Table 4:** The detailed information of the activation curve

Study	Type	Dose	Number	Half of the activating voltage (mV)		Slope factor	
				Pre-treatment	Post-treatment	Pre-treatment	Post-treatment
Xiangyu Li et al. 2013	I_k_	5 μg/g	6	23.65 ± 0.65	28.19 ± 0.57*	6.09 ± 0.56	5.14 ± 0.51*
Ming Cao et al. 2010	I_Ca-L_	10 μg/g	5	−5.47 ± 0.50	−2.77 ± 0.49*	4.68 ± 0.39	4.50 ± 0.40
Tao Yang et al. 2009	I_Na_	5 μg/g	6	−43.65 ± 0.98	−40.25 ± 1.01*	5.63 ± 0.75	5.03 ± 0.80*
Langjie Hu et al. 2009	I_to_	6 μg/g	5	36.06 ± 1.79	34.79 ± 3.03	22.97 ± 1.49	30.79 ± 2.90

*means *P* < 0.05

**Table 5:** The detailed information of the inactivation curve

Study	Type	Dose	Number	Half of the activating voltage (mV)		Slope factor	
				Pre-treatment	Post-treatment	Pre-treatment	Post-treatment
Xiangyu Li et al. 2013	I_k_	5 μg/g	6	−64.46 ± 1.02	−82.84 ± 1.27*	14.40 ± 1.13	13.35 ± 1.06*
Ming Cao et al. 2010	I_Ca-L_	10 μg/g	5	−20.82 ± 0.48	−29.44 ± 1.03*	6.16 ± 0.43	11.05 ± 0.86*
Tao Yang et al. 2009	I_Na_	5 μg/g	6	−100.92 ± 0.68	−111.20 ± 0.86*	10.33 ± 0.62	11.33 ± 0.74*
Langjie Hu et al. 2009	I_to_	6 μg/g	5	−33.74 ± 0.48	−40.54 ± 0.70*	5.00 ± 0.40	8.42 ± 0.62*

Note the following: * means *P* < 0.05

The Nav1.5-HEK cell has only individual sodium channels, encodes mouse cDNA and is expressed in human embryonic kidney (HEK) cells. Three studies confirmed the antiarrhythmic effects of the volatile oil based on the Nav1.5-HEK cell [32–34]. As the results showed, the volatile oil of NC had an obvious inhibitory effect on sodium membrane potential in a dose-dependent manner. In addition, the volatile oil of NC could accelerate inactivation and inhibit the activation of sodium channels. Current-voltage, conductance-voltage and the current-frequency relationship of the Nav1.5- HEK cell were also affected by the volatile oil of NC.

Three studies reported the antiarrhythmic functions of NC and its active compounds in animal experiments [23–25] (Table 6). In the first study, 0.002% aconitine, 1 mL/kg, was used to treat rats in a model group and a Nardosinone group to establish a tachyarrhythmia animal model [23]. The Nardosinone group was treated by 100 μg/g of nardosinone via intraperitoneal injection and the normal and tachyarrhythmia groups were given the same volume of saline. Compared with the normal group, the model group exhibited significant increases in heart rate (HR), left ventricular end-diastolic pressure (LVEDP), the heart coefficient, Ca^2+^ level, cyclic adenosine monophosphate (cAMP) and protein kinase A (PKA). In contrast, the levels of left ventricular systolic pressure (LVSP), +dp/dt_max_, and -dp/dt_max_ decreased. Compared with the model group, the above indexes were obviously improved in the Nardosinone group. In addition, the differences in cAMP and PKA between the normal group and the Nardosinone group were subtle.

**Table 6:** The detailed information of the animal experiment

Study	Species	Weight(g)	Random	Groups	Outcome measure
Peng Jian et al. 2015	Wistar rats	200 ± 20	Random digits table	The normal group (*n* = 20) The tachyarrhythmia group (*n* = 20) The Nardosinone group (*n* = 20)	HR; Heart coefficient; LVSP; LVEDP; +dp/dt_max_; −dp /dt_max_; cAMP; PKA
Jing Zhang et al. 2014	SD rats	280–310	Not mentioned	The control group (*n* = 24) The metoprolol group (*n* = 24) The NC group (*n* = 24)	PVCs; VTs; VFs; HR; MAP; P-R interval; QRS interval; Q-T interval; Cx43; Infarct size of the left ventricular
Ping Zhou et al. 2007	Wistar rats	250 ± 50	Not mentioned	The control group-1 (*n* = 6) The control group-2 (*n* = 6) The volatile oil of NC group-1 (*n* = 20) The volatile oil of NC group-2 (*n* = 20)	ERP; ERP/RR

*HR* Heart rate; Heart coefficient = Heart weight (mg)/rat weight(g); *LVSP* Left ventricular systolic pressure, *LVEDP* Left ventricular end-diastolic pressure, *cAMP* Cyclic adenosine monophosphate, *PKA* Protein kinase A, *PVCs* Premature ventricular contractions, *VTs* Ventricular tachycardias, *MAP* Mean arterial pressure; *VFs* Ventricular fibrillations, *Cx43* Connexin 43, *ERP* Effective refractory period

The goal of the second animal experiment was to investigate the mechanisms of NC on spontaneous ventricular arrhythmias in rats with AMI [24]. Compared with the controls, metoprolol and NC could decrease the ventricular fibrillation (VF) incidence, the cumulative number of premature ventricular contractions (PVCs) and ventricular tachycardias (VTs), and the infarct size of the left ventricular tissue. However, no significant differences were observed in the above indexes between the metoprolol and NC groups. Metoprolol significantly prolonged the P-R interval and decreased the HR and mean arterial pressure (MAP). However, no significant differences were found in the QRS or the Q-T intervals between the control and treatment groups. NC also decreased HR, but it produced no significant changes in MAP, the P-R interval, the QRS interval or the Q-T interval. Compared with NC, metoprolol significantly prolonged the P-R interval. In addition, the results from immunohistochemistry, Western blot, and RT-PCR showed that the protein expression of Connexin 43 (Cx43) in the control group was significantly lower than that in the metoprolol and NC groups.

The third experiment presented the validated functions of the volatile oil of NC on the ventricular effective refractory period (ERP) and compared its therapeutic effects between local infiltration and airway inhalation at different time points [25]. This study showed that the ERP could be remarkably prolonged by the volatile oil of NC and that the best intervention time point was 15–45 min. For the drug delivery methods, airway inhalation was more effective than local infiltration.

### Assessment of the risk of bias for animal studies

There were five animal articles in our manuscript [19, 20, 23–25]. According to SYRCLE’s risk of bias tool, we found that the detailed information of the studies was unclear regarding selection bias, performance bias, detection bias, attrition bias, reporting bias and so forth. Only one study [23] mentioned a random number table, and the others did not report the random allocation sequence generation methods. In addition, all studies described the completeness of the outcome data for each main outcome, as shown in Table 7.

**Table 7:** Assessment of the risk of bias for animal studies

Studies	1	2	3	4	5	6	7	8	9	10
Tao Yang et al. 2012	Unclear	Yes	Unclear	Unclear	Unclear	Unclear	Unclear	Yes	Unclear	Unclear
Rajakannu Subashini et al. 2006	Unclear	Yes	Unclear	Unclear	Unclear	Unclear	Unclear	Yes	Unclear	Unclear
Peng Jian et al. 2015	Yes	Yes	Unclear	Unclear	Unclear	Unclear	Unclear	Yes	Unclear	Unclear
Jing Zhang et al. 2014	Unclear	Yes	Unclear	Unclear	Unclear	Unclear	Unclear	Yes	Unclear	Unclear
Ping Zhou et al. 2007	Unclear	Yes	Unclear	Unclear	Unclear	Unclear	Unclear	Yes	Unclear	Unclear

Selection bias (sequence generation); 2. Selection bias (baseline characteristics); 3. Selection bias (allocation concealment); 4. Performance bias (random housing); 5. Performance bias (blinding); 6. Detection bias (random outcome assessment); 7. Detection bias (blinding); 8. Attrition bias (incomplete outcome data); 9. Reporting bias (selective outcome reporting); 10. Other (other sources of bias)

## Discussion

NC is a traditional Chinese medicine that has beneficial effects on myocardial protection through the inhibition of myocardial apoptosis, inflammation and oxidative stress [19–22]. It has also been shown to modulate ion channels against cardiac arrhythmias [26–34]. Ion channels represent a class of special hydrophilic protein channels on cytomembranes and one of their important functions is to generate bioelectricity [48, 49]. The movement of ions, such as sodium, potassium, calcium, and chloride, through the myocardial cell membrane forms the basis of electrical activity in myocardial cells. If ion movement across cell membranes is disorderly, cardiac arrhythmias can manifest [50]. Therefore, searching for new effective modalities to regulate ion channels is challenging. Many current studies have focused on interventions that can treat and prevent cardiac arrhythmia.

The generation of myocardial cell action potentials is the basis of cardiac electrical activity, which includes five phases (from 0 to 4) [51]. Depolarization from the SA node brings the membrane potential to the threshold, opening the voltage-gated sodium channels and giving rise to the peak current of Na + and the rapid upstroke (phase 0) of the cardiac action potential. Inactivation of the sodium channel and activation of I_to_ are the predominant contributors to the partial membrane repolarization in the first phase. Phase 2, the videlicet plateau, is a long phase due to the delicate balance between the inward currents (mostly I_Ca-L_) and the outward currents (mostly I_k_). As the inward currents (I_Ca-L_) become inactivated, the outward currents (I_k)_ predominate, causing further repolarization and bringing the membrane potential towards the potassium equilibrium potential (phase 3). Then, the membrane potential returns to its resting potential after full repolarization during phase 4, which depends on numerous components such as the Na^+^ − K^+^ pump, the Na^+^ − Ca^2+^ exchanger and the Ca^2+^ pump to recover the normal concentration gradient of the myocardial cell membrane.

The role of I_k1_ is to influence the resting potential of the myocardial cell membrane and repolarization (phases 2 and 3) in fast response myocardial cells. The volatile oil of NC can inhibit I_k1_ in a concentration-dependent manner and decrease I_k1_ density under different voltage regulations in isolated ventricular myocytes in rats [23]. Therefore, NC may depend on a decreased I_k1_ to produce its antiarrhythmic effects. Interestingly, a large proportion of antiarrhythmic drugs do not regard I_k1_ as a main therapeutic target. The reason may involve two aspects. On one hand, a highly selective I_K1_ blocker or agonist is not available. On the other hand, the proarrhythmic or antiarrhythmic effects of I_K1_ are not very clear if I_K1_ is inhibited or blocked. More evidence is essential to confirm the regulatory mechanism of I_k1_ on cardiac arrhythmias.

I_to_, the main current responsible for the early rapid repolarization (phase 1) in fast response cells, has been proven to exist extensively in myocardial cells, especially in atrial and ventricular myocytes in mammals. It has a significant effect on the shape and the duration of the cardiac action potential [52]. In addition, the I_to_ is characterized by a transmural gradient in current density across the ventricular myocardium that leads to significant differences in cardiac action potentials between the endocardium and the epicardium [53]. This distribution gives rise to repolarization heterogeneity and is probably responsible for the main pathogenesis of ventricular tachycardia and ventricular fibrillation.

I_k_ is able to mediate repolarization in fast (phase 2, 3) and slow (phase 3) response myocardial cells. In addition, a degenerative I_k_ partially participates in the spontaneous depolarization (phase 4) of Purkinje cells and sinoatrial node cells [54]. Therefore, I_k_ is closely related to action potential duration (APD) and the effective refractory period (ERP). If I_k_ occurs abnormally, the excitability and conductibility of myocardial cells could change. Then, all kinds of cardiac arrhythmias can manifest due to abnormal rhythmicity, reentry and triggered activity.

Na^+^ channels are widely distributed in myocardial cells in mammals and mediate the excitability and conduction of the heart. There are two sub-types of sodium channels. Voltage-dependent sodium channels (I_Na_), i.e., the fast sodium current, are primarily responsible for depolarization (phase 0) in myocardial action potentials [55]. The slow sodium current channels (I_Na-S_) maintain balance in the plateau [56]. In addition, the pacemaker current (I_f_) has a crucial role in the spontaneous depolarization of Purkinje and sinoatrial node cells, and the Na^+^ current constitutes a large proportion of all ion currents [57]. Therefore, disorder of the Na^+^ current can promote the occurrence of cardiac arrhythmia.

There are three types of calcium channels in myocardial cells, i.e., B-type, L-type, and T-type [58]. L-type and T-type calcium channels plays major roles in myocardial electrophysiological activity. The activated L-type calcium channel is capable of generating a slow inward calcium current (I_Ca-L_), which is the ionic basis of ventricular cardiac action potentials during phase 2 [59]. T-type calcium channels mainly exist in cardiac autonomic cells, such as sinoatrial node cells, and are activated to affect the pacemaker activity of the heart through a slow inward calcium current (I_Ca-T_) [60]. An abnormal Ca^2+^ current can easily cause early after depolarization (EAD) and delayed after depolarization (DAD), which contribute to the main pathogenesis of cardiac arrhythmia [61].

Cardiac arrhythmias can be clinically divided into tachycardic arrhythmias and bradycardiac arrhythmias. Bradycardiac arrhythmias are treated by pharmacological agents, such as atropine, or by the installation of a temporary or permanent pacemaker to decrease haemodynamic disturbances [62]. Most antiarrhythmic drugs act as various ion channel or receptor blockers and are mainly used for tachycardic arrhythmias. According to the electrophysiological characteristics of these medications, antiarrhythmic agents can be divided into four categories, i.e., Na^+^ channel, β-receptor, K^+^ channel and Ca^2+^ channel blockers [63].

In terms of the main currents involved in action potentials (I_k_, I_to_, I_Ca-L_ and I_Na_), NC and its active compounds could inhibit these currents in a dose-dependent manner and decrease current density under different voltage regulation. In addition, NC and its active compounds not only suppress the activation but also promote the inactivation of ion channels in isolated ventricular myocytes in rats and rabbits. Similar to the mechanisms of some Western antiarrhythmic medicines, NC and its compounds exhibit antiarrhythmic effects by restraining these ion channels or prolonging the ERP.

## Conclusion

*Nardostachys chinensis* has certain cardioprotective and antiarrhythmic effects in animal and cell experiments by inhibiting myocardial apoptosis, the inflammation reaction, and oxidative stress and by modulating several ion channels.

## Study limitations

NC is one of the traditional Chinese medicines used for the treatment of cardiac diseases, especially cardiac arrhythmia. The therapeutic mechanism of NC and its active compounds was demonstrated in animal and cell experiments. However, many limitations and deficiencies remain. The details are shown as follows: (i) There are not enough studies to confirm the cardioprotective and antiarrhythmic effects of NC or its active compounds. (ii) The studies mainly involved isolated ventricular myocytes or Nav1.5-HEK cells, and other rhythmic cells, such as sinoatrial node cells and Purkinje cells, were not studied, leading to a lack of information on the mechanism of NC. (iii) The volatile oil and the extract of NC are compounds in NC, so it is necessary to determine the monomer composition to verify the therapeutic targets of NC. (iv) The isolated ventricular myocytes were derived from healthy rats or rabbits. Therefore, whether NC has a beneficial role in myocardial cells in animals exhibiting cardiac arrhythmia remains unclear. (v) Most studies are self-controlled, which may lead to low quality in the literature. (vi) Currently, no research studies have reported the effects of NC on I_Ca-T_, I_Na-S_ or I_f_. (vii) The studies show that NC has inhibitory effects on tachycardic arrhythmia. Therefore, whether NC is appropriate for bradycardiac arrhythmia remains uncertain.

## Future perspectives

Although the above studies focused heavily on the mechanism of NC via cell and animal experiments, there is a lack of information regarding the effects of NC in cardiac diseases at the molecular level. In addition, the occurrence of diseases is due to imbalances in gene expression, so it is crucial to restore balance through a variety of interventions. Further relevant research in genomics, transcriptomics, metabolomics and proteomics would need to be conducted for a better understanding of this mechanism. In addition, pharmacological treatments for cardiac arrhythmia are usually administered orally or intravenously in clinical settings. However, cardiac arrhythmias are often paroxysmal. Oral drugs are slow to treat, and intravenous drugs are typically only used in hospitals. These problems are worth consideration. The volatile oil of NC in the form of a nasal spray may be a viable alternative modality in the treatment of cardiac arrhythmia.

## Abbreviations

ALT: Alanine aminotransaminase
AMI: Acute myocardial infarction
Ang II: Angiotensin II
ANP: Atrial natriuretic peptide
APD: Action potential duration
ARE: Antioxidant response element
AST: Aspartate aminotransaminase
BNP: B-type natriuretic peptide
CAM: Complementary and/or alternative medicine
cAMP: Cyclic adenosine monophosphate
CAST: Cardiac Arrhythmia Suppression Trial
CAT: Catalase
CK-MB: Creatine kinase-MB
CPK: Creatine phosphokinase
cTnT: Cardiac troponin T
CVDs: Cardiovascular diseases
Cx43: Connexin 43
DAD: Delayed after depolarization
EAD: Early after depolarization
ERK: Extracellular signal-regulated kinase
ERP: Effective refractory period
GPx: Glutathione peroxidase
GST: Glutathione-S-transferase
G-V: Conductance-voltage relationship
HEK: Human embryonic kidney
HR: Heart rate
I/R: Ischaemia-Reperfusion
I_Ca-L_: L-type calcium current
I_k_: Delayed rectifier potassium current
I_k1_: Inward rectifier potassium current
IL-6: Interleukin-6
I_Na_: Sodium current
I_to_: Transient outward potassium current
I-V: Current-voltage relationship
LDH: Lactate dehydrogenase
LPO: Lipid peroxide
LVEDP: Left ventricular end-diastolic pressure
LVSP: Left ventricular systolic pressure
MAP: Mean arterial pressure
MEK: Mitogen-activated protein kinase
NC: *Nardostachys chinensis*
PI3K: Phosphatidylinositol 3-kinase
PKA: Protein kinase A
PVCs: Premature ventricular contractions
RCTs: Randomized controlled trials
ROS: Reactive oxygen species
SOD: Superoxide dismutase
tBHP: Tert-butyl hydroperoxide
TCM: Traditional Chinese medicine
TNF-ɑ: Tumour Necrosis Factor
VFs: Ventricular fibrillations
VPB: Ventricular premature beat
VTs: Ventricular tachycardias
β-MHC: β-myosin heavy chain

## References

[1] Riaz M, Zia-Ul-Haq M, Saad B. The Role of Anthocyanins in Health as Antioxidant, in Bone Health and as Heart Protecting Agents In: Anthocyanins and Human Health: Biomolecular and Therapeutic Aspects: Springer International Publishing; 2016.

[2] Yang B, Benzhi C (2010). Advances in the study of arrhythmogenic mechanisms. Journal of International Pharmaceutical Research..

[3] Nattel S, Andrade J, Macle L, Rivard L, Dyrda K, Mondesert B (2014). New directions in cardiac arrhythmia management: present challenges and future solutions. Can J Cardiol..

[4] Antzelevitch C, Burashnikov A (2011). Overview of Basic Mechanisms of Cardiac Arrhythmia. Card Electrophysiol Clin..

[5] Yousuf O, Chrispin J, Tomaselli GF, Berger RD (2015). Clinical management and prevention of suddencardiac death. Circulation Research.

[6] Lüderitz B (2002). History of cardiac rhythm disorders. Z Kardiol..

[7] Sanguinetti MC, Bennett PB (2003). Antiarrhythmic drug target choices and screening. Circ Res..

[8] Preliminary report: effect of encainide and flecainide on mortality in a randomized trial of Arrhythmia suppression after myocardial infarction. The Cardiac Arrhythmia Suppression Trial (CAST) Investigators.1989;321(6):406-12. 10.1056/NEJM198908103210629 2473403

[9] Nautiyal BP, Chauhan RS, Prakash V, Purohit H, Nautiyal MC (2003). Population studies for the evaluation of germplasm and threat status of the alpine medicinal herb Nardostachys jatamansi. Journal of Statistical Physics..

[10] The Pharmacopoeia of the People’s Republic of China. Pt 1. 2015:86.

[11] Yuan jie, Han Zucheng, Wang Min, Chen Qing. The research situation of *Nardostachys chinensis*. Chinese Journal of Ethnomedicine and Ethnopharmacy. 2012;21(16), 57-59.

[12] Itokawa H, Masuyama K, Morita H, Takeya K (1993). Cytotoxic sesquiterpenes from *Nardostachys chinensis*. Chem Pharm Bull (Tokyo)..

[13] Li L, Wang Y (2010). The study on the active components of *Nardostachys chinensis*. Journal of Capital Normal University (Natural Science Edition)..

[14] Zhibin C, Fen L, Jiakun H, Yongping H (2008). Pharmacological screening of antiarrhythmic fractions from Nardostachyson. Journal of Southwest University for Nationalities- Natural Science Edition..

[15] Hua W, Gao RL, Zhao BC, Wang J, Chen XH, Cai C (2015). The Efficacy and Safety of Wenxin Keli in Patients with Frequent Premature Ventricular Contractions: A Randomized, Double-blind, Placebo-controlled, Parallel-group, Multicenter Trial. Chin Med J (Engl)..

[16] Hu D, Barajas-Martínez H, Burashnikov A, Panama BK, Cordeiro JM, Antzelevitch C (2016). Mechanisms underlying atrial-selective block of sodium channels by Wenxin Keli: Experimental and theoretical analysis. Int J Cardiol.

[17] Gu CH, Wu YL, Tian SY, Gao X, Qi X, Jia Z (2005). Effect of shensong yangxin capsule on ventricular premature beat and cardiovascular autonomic nervous function in patients with coronary heart disease. Zhong guo Zhong Xi Yi Jie He Za Zhi..

[18] Hooijmans CR, Rovers MM, de Vries RB, Leenaars M, Ritskes-Hoitinga M, Langendam MW (2014). SYRCLE’s risk of bias tool for animal studies. BMC Med Res Methodol..

[19] Yang T, Yuan Y, Xu M, Xiaoxia Z, Chuanhua B, Xu T (2012). Effects of the volatile oil of *Nardostachys chinensis* administered at different time points on myocardial ischemia- reperfusion injury in rats. Chinese Journal of Hospital Pharmacy..

[20] Subashini R, Yogeeta S, Gnanapragasam A, Devaki T (2006). Protective effect of Nardostachys jatamansi on oxidative injury and cellular abnormalities during doxorubicin-induced cardiac damage in rats. J Pharm Pharmacol..

[21] Maiwulanjiang M, Chen J, Xin G, Gong AG, Miernisha A, Du CY (2014). The volatile oil of Nardostachyos Radix et Rhizoma inhibits the oxidative stress-induced cell injury via reactive oxygen species scavenging and Akt activation in H9c2 cardiomyocyte. J Ethnopharmacol..

[22] Meng Du, Kun Huang, Lu Gao, Yang L, Wang WS, Wang B, et al. Nardosinone Protects H9c2 Cardiac Cells from Angiotensin II-induced Hypertrophy. J Huazhong Univ Sci Technolog Med Sci. 2013;33(6):822-826. 10.1007/s11596-013-1205-9 24337842

[23] Ping Z. Academic dissertation. The Effects of the Volatile Oil of Gan Song on Effective Refractory Period and value of corrected ERP of Myocardium of Rats. 2008;

[24] Peng J, Qinghai L, Lihua F (2015). Experimental study on the inhibitory effect of nardosinone on myocardial cell in rats with tachyarrhythmia. The Chinese Journal of Clinical Pharmacology..

[25] Zhang J, Qiang CC, Li WJ, Liu LJ, Lin XX, Cheng YJ (2014). Effects of *Nardostachys chinensis* on Spontaneous Ventricular Arrhythmias in Rats With Acute Myocardial Infarction. J Cardiovasc Pharmacol.

[26] Yuxiang L, Luo J, Yuzhi G, Wang Y, Shuhua Z, Wu Z (2013). The Effect of the volatile oil of *Nardostachys chinensis* on I_k_ and I_k1_ in Isolated Ventricular Myocytes in Rats. Lishizhen Medicine and Materia Medical Research..

[27] Qizhu T, Zhengrong H, Xiteng S, Wang T, Shen D (2004). The Effect of the extract of *Nardostachys chinensis* on Sodium and Calcium Channel in Isolated Ventricular Myocytes in Rats. Chinese Journal of Cardiology..

[28] Ming C, Yuzhi G, Lu J, Wang Y, Shuhua Z, Wu Z (2010). The Effect of the volatile oil of *Nardostachys chinensis* on L-Type Calcium Channel in Isolated Ventricular Myocytes in Rats. Lishizhen Medicine and Materia Medical Research..

[29] Yuanwei L, Guo J, Ping Z, Jiwen L, Chun L (2009). The effects of nardostachys chinensis batal extract on the sodium current and transient outward potassium current of rat ventricular myocytes. Chinese Journal of Cardiac Pacing and Electrophysiology..

[30] Yang T, Yuzhi G, Luo J, Hu L, Shuhua Z (2010). The Effect of the volatile oil of *Nardostachys chinensis* on Sodium Channel in Isolated Ventricular Myocytes in Rats. Lishizhen Medicine and Materia Medical Research..

[31] Hu L, Yuzhi G, Luo J, Yang T, Shuhua Z (2009). The Effect of the volatile oil of *Nardostachys chinensis* on Transient outward potassium current in Isolated Ventricular Myocytes in Rats. Lishizhen Medicine and Materia Medical Research..

[32] Yuzhi G, Zhiting W, Shuahua Z, Guotai S, Yeh Jay Z (2009). Blockade of current of Na_v_1.5 in HEK cells depended by the voltage and concentration of volatile oil of Nardostachy Chinesis Btala. Pharmacology and Clinics of Chinese Materia Medical..

[33] Yuzhi G, Zhiting W, Langji H, Yeh Jay Z (2009). The Effects of the Volatile Oil of Nardosta chychinesis Batal on Na^+^ Current of the Cardiac Sodium Channels of Rats and Na_v_1.5 in HEK Cells. Lishizhen Medicine and Materia Medical Research..

[34] Yanyang L, Yuzhi G, Yeh JZ (2013). The effects of Volatile 0il of Nardostachys Chinesis Batal (VONCB) on frequency-dependent block of Na^+^ channels (Na_v_1.5) in human embryonic kidney (HEK) cells. Lishizhen Medicine and Materia Medical. Research..

[35] Kovacic P, Somanathan R (2012). Redox process in neurodegenerative disease involving reactive oxygen species. Current Neuropharmacology..

[36] Ichihara S (2013). The pathological roles of environmental and redox stresses in cardiovascular disease. Environmental Health and Preventive Medicine..

[37] Heineke J (2006). Molkentin Jd. Regulation of cardiac hypertrophy by intracellular signaling pathways. Nat Rev. Mol Cell Biol..

[38] Hunyady L, Turu G (2004). The role of the AT_1_ angiotensin receptor in cardiac hypertrophy: angiotensin II receptor or stretch sensor?. Trends Endocrinol Metab..

[39] Hung J, Teng TH, Finn J, Knuiman M, Briffa T, Stewart S (2013). Trends from 1996 to 2007 in incidence and mortality outcomes of heart failure after acute myocardial infarction: a population-based study of 20812 patients with first acute myocardial infarction in Western Australia. J Am Heart Assoc.

[40] Rezkalla SH, Dharmashankar KC, Abdalrahman IB, Kloner RA (2010). No-reflow phenomenon following percutaneous coronary intervention for acute myocardial infarction: incidence, outcome, and effect of pharmacologic therapy. J Interv Cardiol.

[41] Domburg RT, Hendriks JM, Kamp O, Smits P, Mv M, Schenkeveld L (2012). Three life years gained after reperfusion therapy in acute myocardial infarction: 25-30 years after a randomized controlled trial. Eur J Prev Cardiol..

[42] Gross GJ, Auchampach JA (2007). Reperfusion injury: does it exist?. J Mol Cell Cardiol..

[43] O'Donoghue ML, Glaser R, Cavender MA, Aylward PE, Bonaca MP, Budaj A (2016). Effect of Losmapimod on Cardiovascular Outcomes in Patients Hospitalized With Acute Myocardial Infarction: A Randomized Clinical Trial. JAMA..

[44] Iliodromitis K, Farmakis D, Andreadou I, Zoga A, Bibli SI, Manolaki T (2013). Various models of cardiac conditioning in single or sequential periods of ischemia: comparative effects on infarct size and intracellular signaling. Int J Cardiol..

[45] Lefrak EA, Pitha J, Rosenheim S, Gottlieb JA (1973). A clinicopathologic analysis of Adriamycin cardiotoxicity. Cancer..

[46] Nagi MN, Mansour MA (2000). Protective effect of Thymoqui-none against Doxorubicin-induced cardiotoxicity in rats: a possible mechanism of protection. Pharmacol. Res..

[47] Yoon J, Leblanc N, Zaklit J, Vernier PT, Chatterjee I, Craviso GL (2016). Enhanced Monitoring of Nanosecond Electric Pulse-Evoked Membrane Conductance Changes in Whole-Cell Patch Clamp Experiments. J Membr Biol..

[48] Decker KF, Rudy Y (2010). Ionic mechanisms of electrophysiological heterogeneity and conduction block in the infarct border zone. Am J Physiol Heart Circ Physiol..

[49] Gima K, Rudy Y (2002). Ionic current basis of electrocardiographic waveforms: a model study. Circ Res..

[50] O'Hara T, Rudy Y (2012). Arrhythmia formation in subclinical (“silent”) long QT syndrome requires multiple insults: quantitative mechanistic study using the KCNQ1 mutation Q357R as example. Heart Rhythm..

[51] Tse G (2016). Mechanisms of cardiac arrhythmias. J Arrhythm..

[52] Barry DM, Xu H, Schuessler RB, Nerbonne JM (1998). Functional knockout of the transient outward current, long-QT syndrome, and cardiac remodeling in mice expressing a dominant-negative Kv4 alpha subunit. Circ Res..

[53] Perrin MJ, Adler A, Green S, Al-Zoughool F, Doroshenko P, Orr N (2014). Evaluation of genes encoding for the transient outward current (Ito) identifies the KCND2 gene as a cause of J-wave syndrome associated with sudden cardiac death. Circ Cardiovasc Genet.

[54] Ke Y, Lei M, Collins TP, Rakovic S, Mattick PA, Yamasaki M (2007). Regulation of L-type calcium channel and delayed rectifier potassium channel activity by p21-activated kinase-1 in guinea pig sinoatrial node pacemaker cells. Circ Res.

[55] Lape R, Nistri A (2001). Characteristics of fast Na(+) current of hypoglossal motoneurons in a rat brainstem slice preparation. Eur J Neurosci.

[56] Guo D, Young L, Wu Y, Belardinelli L, Kowey PR, Yan GX (2010). Increased late sodium current in left atrial myocytes of rabbits with left ventricular hypertrophy: its role in the genesis of atrial arrhythmias. Am J Physiol Heart Circ Physiol..

[57] Xue T, Siu CW, Lieu DK, Lau CP, Tse HF, Li RA (2007). Mechanistic role of I(f) revealed by induction of ventricular automaticity by somatic gene transfer of gating-engineered pacemaker (HCN) channels. Circulation..

[58] Rosenberg RL, Hess P, Tsien RW (1988). Cardiac calcium channels in planar lipid bilayers. L-type channels and calcium-permeable channels open at negative membrane potentials. J Gen Physiol..

[59] Yamaoka K, Kameyama M (2003). Regptulation of L-type Ca2+ channels in the heart: overview of recent advances. Mol Cell Biochem..

[60] Ono K, Iijima T (2010). Cardiac T-type Ca (2+) channels in the heart. J Mol Cell Cardiol..

[61] Asakura K, Cha CY, Yamaoka H, Horikawa Y, Memida H, Powell T, Amano A, Noma A (2014). EAD and DAD mechanisms analyzed by developing a new human ventricular cell model. Prog Biophys Mol Biol..

[62] Willich T, Goette A (2014). Bradycardic arrhythmias. Dtsch Med Wochenschr..

[63] Vaughan Williams EM (1992). Classifying antiarrhythmic actions: by facts or speculation. J Clin Pharmacol..

